# Conservation planning under uncertainty in urban development and vegetation dynamics

**DOI:** 10.1371/journal.pone.0195429

**Published:** 2018-04-05

**Authors:** David Troupin, Yohay Carmel

**Affiliations:** 1 Faculty of Architecture and Town Planning, Technion–Israel Institute of Technology, Haifa, Israel; 2 Faculty of Civil and Environmental Engineering, Technion–Israel Institute of Technology, Haifa, Israel; Pennsylvania State University, UNITED STATES

## Abstract

Systematic conservation planning is a framework for optimally locating and prioritizing areas for conservation. An often-noted shortcoming of most conservation planning studies is that they do not address future uncertainty. The selection of protected areas that are intended to ensure the long-term persistence of biodiversity is often based on a snapshot of the current situation, ignoring processes such as climate change. Scenarios, in the sense of being accounts of plausible futures, can be utilized to identify conservation area portfolios that are robust to future uncertainty. We compared three approaches for utilizing scenarios in conservation area selection: considering a full set of scenarios (all-scenarios portfolio), assuming the realization of specific scenarios, and a reference strategy based on the current situation (current distributions portfolio). Our objective was to compare the robustness of these approaches in terms of their relative performance across future scenarios. We focused on breeding bird species in Israel’s Mediterranean region. We simulated urban development and vegetation dynamics scenarios 60 years into the future using DINAMICA-EGO, a cellular-automata simulation model. For each scenario, we mapped the target species’ available habitat distribution, identified conservation priority areas using the site-selection software MARXAN, and constructed conservation area portfolios using the three aforementioned strategies. We then assessed portfolio performance based on the number of species for which representation targets were met in each scenario. The all-scenarios portfolio consistently outperformed the other portfolios, and was more robust to ‘errors’ (e.g., when an assumed specific scenario did not occur). On average, the all-scenarios portfolio achieved representation targets for five additional species compared with the current distributions portfolio (approximately 33 versus 28 species). Our findings highlight the importance of considering a broad and meaningful set of scenarios, rather than relying on the current situation, the expected occurrence of specific scenarios, or the worst-case scenario.

## Introduction

Systematic Conservation Planning is a framework for optimally locating, selecting, prioritizing, and designing conservation area portfolios, in which biodiversity is well-represented, protected, and able to persist [1–3]. Over the past several decades the systematic conservation planning framework has been increasingly utilized throughout the world in conservation case studies and as a decision making support tool [1,3,4]. Central questions in conservation planning are how to prioritize areas for protection and where to allocate resources and efforts. Typically, systematic conservation planning aims to protect multiple features of biodiversity, using species and habitat types as the targeted conservation features [5].

An often-noted shortcoming of most conservation planning studies is that they do not account for future uncertainty [2,3,6–8]. Uncertainty is an inseparable issue when modeling processes and making predictions regarding the future [9,10]. One source of future uncertainty arises from the difficulty of predicting the outcome of processes and their impact. Such uncertainty regarding the future can be the result of different types of epistemic uncertainty (uncertainty about facts) [9,11]: inherent randomness and natural variation of processes, data errors, limited knowledge of processes, and the limited ability of models to represent reality [2,9]. In most conservation planning studies, the spatial distributions of species and other biodiversity features are often assumed to be known and constant [2,8,11]. Thus, the selection of protected areas that are intended to ensure the long-term persistence of biodiversity has often been based on a snapshot of the past or current situation. In this study our goal was to address this gap of dealing with future uncertainty in spatial distributions of species in conservation planning. We focus on two major land cover change processes in Israel's Mediterranean region: vegetation dynamics and urban development, and the identification of conservation priority areas for breeding birds under the uncertainty that results from different scenarios of these processes.

Scenario planning is one of the widely-used tools for dealing with uncertainty, by exploring a range of future alternatives and consequences of associated decisions [12,13]. A scenario in this context is an account of a plausible future and typically several contrasting scenarios are applied in order to explore the uncertainty surrounding the future consequences of a decision. Until recent years, scenario planning had been underutilized in conservation planning, and it was often noted that the plurality of futures should be acknowledged and that uncertainties about future changes should become a more central concern in this field [10,13–17]. In recent years, several studies have pioneered the incorporation of future scenarios and their associated uncertainty in the process of reserve selection [11,18–25].

For the practice of identifying priority areas for conservation through conservation planning, a main challenge is utilizing scenarios in order to deal with uncertainty concerning the future distributions of the conservation targets (commonly species). Climate change and associated scenarios are a major source of uncertainty in this context [10,11,26,27]. Several conservation planning studies have considered climate change uncertainty directly, by modeling distributions and ranges of species under different scenarios of climate change [18,21,28]. Alternatively, it can be considered as a source of uncertainty influencing other processes that affect habitat suitability and loss or dispersal and movement of species, such as succession and disturbance in plant communities [29,30]. Future uncertainty is also present in other processes that influence species and their distributions and habitats–e.g., different forms of anthropogenic disturbance such as deforestation and urban development which have also been addressed in a few studies [31,32].

There are several approaches for integrating information from scenarios in order to construct conservation area portfolios that are robust to uncertainty. One common approach is to identify areas of high conservation priority across multiple scenarios–i.e., concurrently important across scenarios [18]. Others propose that the best approach is to assume the worst-case scenario, in line with the precautionary principle [33,34]. Another possibility is diversification: e.g., either selecting conservation areas that represent the diversity of biophysical conditions [35] or distributing the investment across scenarios [36]. Ando and Mallory [19]went beyond simple diversification, by implementing modern portfolio theory as a means for efficiently selecting conservation priority areas, given the uncertainty associated with climate change scenarios [19]. To date, only few studies [8,19,22] have examined the relative contribution of utilizing scenarios in reserve selection (i.e., to what degree does the use of scenarios improve robustness?) or compared different approaches for integrating information from scenarios in the design of robust conservation area networks.

Our objective in this study was to evaluate, across the range of possible future scenarios, the robustness of the different approaches and the possible tradeoffs that their relative success or failure would entail. Within the framework of our case study on breeding birds in Israel's Mediterranean region under future uncertainty in vegetation dynamics and urban development, we assess whether using scenarios has benefits in comparison to relying solely on current data without projections and compare three approaches for utilizing scenarios in the selection of conservation area portfolios: a strategy that considers a full set of plausible scenarios, a strategy that utilizes a subset of the plausible scenarios (by assuming that a specific scenario will occur), and a reference strategy–planning conservation areas without scenarios, based only on the current situation.

## Materials and methods

### Overview of methodology

This section briefly outlines the methodology we applied. After defining the study area and study species, we obtained for each individual species a map of its distribution range (from bird and endangered species atlases) and determined its preferred habitat types based on expert knowledge. By overlaying the distribution range maps and the preferred habitat maps we produced maps of current available habitat within the distribution range for each species. We then ran the software program MARXAN (Version 2.4) [37] using these maps as the input. MARXAN is a site-selection software. It is used to identify conservation priority areas. It uses mathematical optimization methods to generate and test spatial configurations that have minimal cost (or area) and meet defined conservation targets (see more detailed explanation on MARXAN in section 2.7). This stage resulted in a conservation area portfolio based on the current situation (current distributions portfolio). We then used a land cover simulation model to simulate scenarios of vegetation dynamics and urban development in the study area for a period of 60 years into the future. We mapped the available habitats for each species under each scenario, and ran MARXAN again, with the maps of the future distribution in each scenario as input. This stage provided us with a conservation area portfolio for each scenario. In addition to the specific conservation area portfolios for each individual scenario, we also selected the highest ranking areas across all scenarios in order to generate a portfolio that is based on the results of each scenario (the all-scenarios portfolio, see detailed explanation in section 2.8). We then examined how each conservation area portfolio (the one based on the current distributions, those based on individual scenarios, and the one based on the combined results of all scenarios) performed (how many species meet their representation target) under the scenarios for which it was designed and under the other scenarios.

### Study area

**Fig 1:**
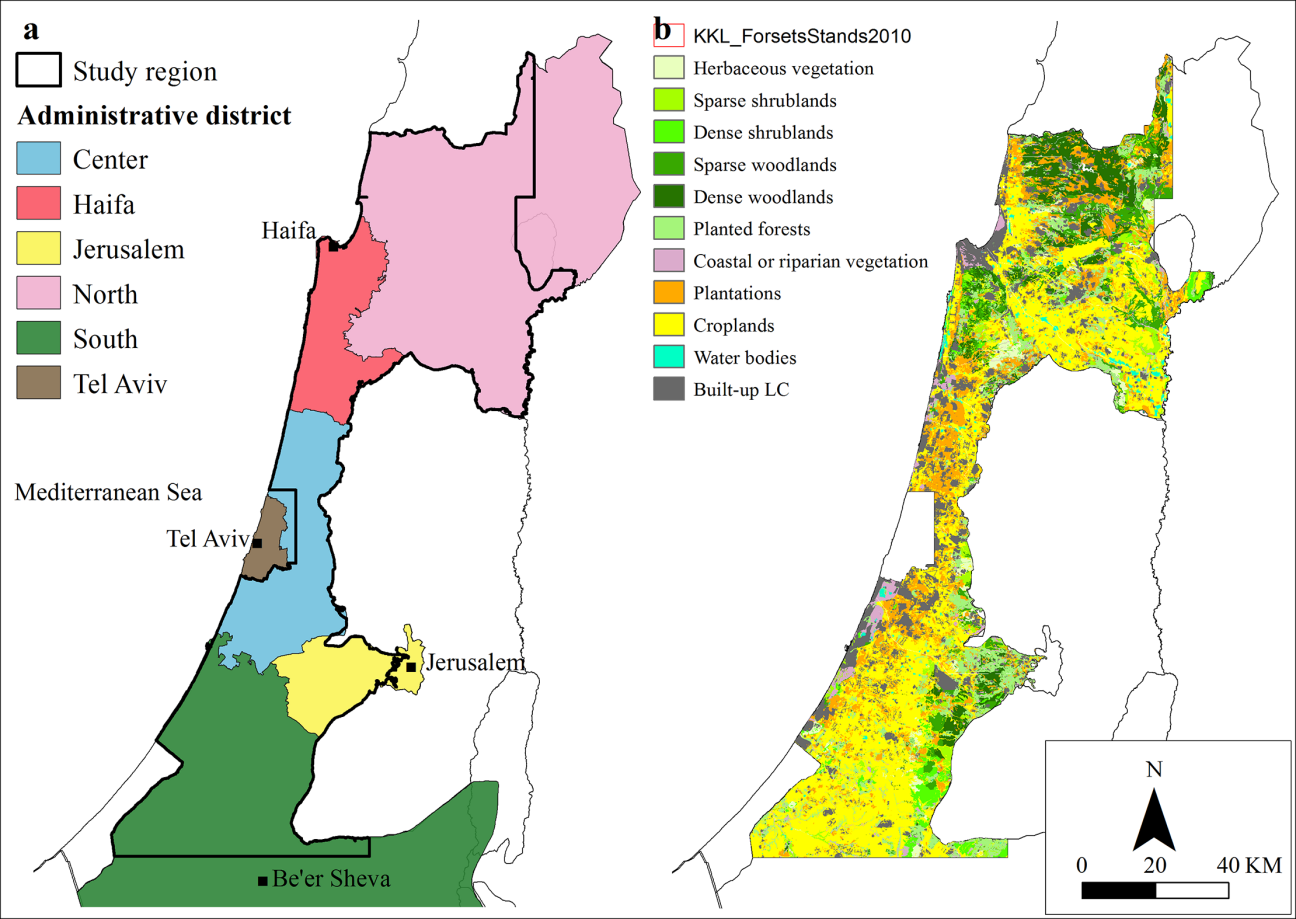
**Geographic sub-regions (a) and land-cover classes (b) in the study area.** For (b) the source year for the land cover classes is as following: built-up land– 2007; plantations and croplands– 2002; planted forests– 2009; and for herbaceous vegetation, shrublands and woodlands (both sparse and dense)– 1995.

Relative to its size, and despite high density of human population and intensive development in recent decades, Israel’s Mediterranean region (Fig 1A) is characterized by a high level of biodiversity, particularly in terms of habitats and rich avifauna [38]. Due to its geographic location, the region is part of several important bird migration routes and serves as a junction for species from several biogeographic regions [38,39]. The study area consists of an area of approximately 7,800 km^2^ which are divided between five different administrative districts: North, Haifa, Center, South and Jerusalem. The Tel Aviv administrative district and the Golan Heights were excluded from the analysis, due to incomplete land-cover data.

### Study species and habitat distribution maps

We focused our analyses on a subset of the breeding bird species that are found in the study area: species that are associated with one or more types of Mediterranean vegetation formations (N = 48, see S1 Table). Some of the species included in the analysis are associated also with agricultural habitats such as croplands and plantations, however the quality of these habitats compared with natural vegetation is often debated [40] and may depend on the type of agricultural practices and other factors. For the purposes of this study, we considered agricultural habitats as unsuitable for the target species, and focused on the selection of natural landscapes that are suitable for establishment as nature reserves.

For each species, we mapped the potential suitable habitat within its distribution range under each of the future scenarios outlined, using the following steps: (1) Definition of distribution range–We defined the distribution range for each species, using the most recent and updated maps available. For 42 of the species included in our analysis this source was the breeding distribution maps from a bird atlas [39] that indicated the population density (high, low, sporadic, localized and historical) of each species at a spatial resolution of 7.5’ x 7.5’ lat/long (corresponding to 11.8 x 13.8 km^2^). From these maps we used the density classes of high, low, sporadic and localized as indicative of each species’ presence. For 6 species, we used more updated distribution range maps that were published in the Red Book of Vertebrates [38]; (2) Definition of suitable habitats–We used land-cover classes as a proxy for breeding bird habitats, since land cover is considered the most dominant factor influencing bird presence at the scale of our study [39]. Three ornithologists ranked the degree of each species’ association (a choice of three levels: strong, moderate, weak/none) to each of the land-cover classes based on their experience and knowledge on each species' breeding and foraging behavior. In cases of discrepancy, we relied on the ranking which received the majority. Using habitat associations of each species and land-cover maps, we produced for each species a map of potential suitable habitats (strong and moderate associations) within the study area; (3) Mapping suitable habitats within each species' distribution range–For each species, we produced a map of suitable habitats within its distribution range, by overlaying the distribution range maps (the product of step 1) with the maps of suitable habitats (the product of step 2), in a manner similar to the method used by Chiozza et al. [41]. We performed this procedure for each scenario as well as for the present-day land-cover map.

Given the paucity of species-specific data on dispersal, we followed Carvalho et al. [18] and assumed that in future scenarios, the presence of each species will be limited to the same grid cells of its current distribution, i.e., no dispersal. As Carvalho et al. [18] pointed out, this is a cautious and conservative approach with regard to dispersal abilities.

As noted above, our purpose was to identify candidate areas for nature reserve establishment. We therefore included only natural land-cover classes as suitable habitats (excluding croplands and agricultural plantations, e.g., orchards, vineyards etc.) and focused our analysis on species that are expected to be influenced by both of the land-cover change processes we modeled: Mediterranean vegetation dynamics and habitat loss as a result of urban development (see section 2.5). The 42 study species in our analysis do not include species that are strongly associated with human settlements and built-up areas, and consist only of species that are associated with one or more of the following vegetation formations: herbaceous vegetation, sparse and dense shrublands, and sparse and dense woodlands.

### Setting representation targets

For each species, we determined a representation target (the area of suitable habitat within its distribution range required to be covered by protected areas), based on its present-day area of available natural habitat [42]. For species with available habitat of < 100 km^2^, we set the representation target at 100% of the area. For widespread species (available habitat > 1,000 km^2^), we set the representation target at 10% of the area. For species with available habitat areas in the intermediate range (100–1,000 km^2^), we determined the representation target by solving the linear equation based on the slope and intercept of the straight line that connects the upper and lower representation target thresholds. Hereinafter, we refer to a species for which the representation target was met as *covered*. We define species for which the representation target was not reached as *underrepresented*.

### Land cover and protected areas

We produced a present-day land cover map for the study area, by integrating data from several sources (Table A in S1 Supporting information, Fig 1B). We approximated the distribution of riparian vegetation and cliffs (both important habitats for many bird species) by overlaying the land-cover map with a 50 m buffer around the running streams and cliff layers, respectively. The resulting map included thirteen land-cover classes (Table B in S1 Supporting information).Protected areas (PAs) included nature reserves and national parks that are managed by the Israel Nature and Parks Authority, and statutory forests which are managed by the Jewish National Fund’s Forest Authority. These organizations provided us with maps of the areas under their management. All layers were provided as vector layers and converted into raster format at a resolution of 50 m.

### Land-cover change model

We simulated urban development and vegetation dynamics in the study area using DINAMICA-EGO [43], a cellular-automata-based simulation model.

The model’s vegetation dynamics component simulated transitions between different formations of Mediterranean vegetation (herbaceous vegetation, sparse and dense shrublands, and sparse and dense woodlands). This process also included two scenarios: (1) vegetation dynamics based on a moderate climate change scenario, corresponding to the IPCC’s B2 scenario [26,44]: in this scenario, vegetation dynamics followed the past trends of succession and fires, which have been reported in other studies on the study area [45–48]; and (2) vegetation dynamics based on a severe climate change scenario, corresponding to the IPCC’s A2 scenario [26,44]: in this scenario, woody vegetation is expected to experience increased mortality and reduced establishment, and the frequency of fire events is expected to increase. These two scenarios result in different distributions and proportions of the various Mediterranean vegetation formations.

For urban development, we simulated two contrasting urban development scenarios (assuming the conversion of both undeveloped agricultural land and non-agricultural land into built-up land) based on two policies: growth management policies (*regulated*) and unregulated development (*unregulated*). Each scenario results in a different spatial development pattern. We simulated each of these scenarios at three different rates of urban development: (1) low: transition probabilities based on those observed between 1998 and 2007 (hereinafter: *baseline transition probabilities*); (2) intermediate: baseline transition probabilities multiplied by 4; and (3) high: baseline transition probabilities multiplied by 8. All values of transition probabilities remained within the range of 0–1 when multiplied for the purposes of the scenarios. Further details on the simulation of urban development can be found in [49] and in S2 Supporting information.

Altogether, we simulated 12 distinct scenarios: two vegetation dynamics (moderate and severe climate change) x two urban development scenarios (regulated and unregulated urban development) x three development magnitudes (low, moderate, and high development rates). The distinct combinations of variables forming each scenario are shown in the three top rows of Table 1. Further details on the simulation framework and the construction of vegetation dynamics scenarios are provided in S2 Supporting information.

**Table 1:** Construction of portfolios.

	Scenarios													
	Vegetation dynamics	S	S	S	S	S	S	M	M	M	M	M	M	t_0_
	Urban development	R	R	R	U	U	U	R	R	R	U	U	U	
	Development rate	L	M	H	L	M	H	L	M	H	L	M	H	
**Portfolios**	Current distributions													x
	All-scenarios	x	x	x	X	x	x	x	x	x	x	x	x	x
	Severe climate change	x	x	x	x	x	x							x
	Moderate climate change							x	x	x	x	x	x	x
	Regulated urban development	x	x	x				x	x	x				x
	Unregulated urban development				x	x	x				x	x	x	x
	Low development	x			x			x			x			x
	Moderate development		x			x			x			x		x
	High development			x			x			x			x	x

The different portfolios we constructed (rows) and the scenarios included in each portfolio (columns). “S” and “M” denote the vegetation dynamics scenarios corresponding to severe and moderate climate change, respectively. “R” and “U” denote regulated and unregulated urban development policy scenarios, respectively. “L”,”M”, and “H” denote the rate of development: low, moderate, and high, respectively. “t_0_” denotes the present-day distribution (current distributions).

### Identifying conservation priority areas for each scenario

After running the scenarios 60 years into the future, and preparing the maps of potential habitat for each species in each scenario, we used the software program MARXAN to identify conservation priority areas for each given scenario after 60 years. MARXAN is a site-selection software that implements simulated annealing (an optimization method) and is widely used for decision support in reserve design and other conservation problems. MARXAN constructs and compares spatial configurations of conservation priority areas (referred to also as portfolios). Each portfolio is represented by an objective function that consists of three components: cost of the planning units included, the boundary length (a representation of fragmentation levels), and the achievement of defined representation targets (penalties are incurred if a targets are not met). MARXAN operates by constructing and testing different portfolios, and attempting to minimize the objective function. In this study we used planning unit area as a surrogate of cost. Given that the software’s algorithm attempts to minimize the cost of the selected priority sites, this served the objective of minimizing the total area needed to meet our defined representation targets. MARXAN enables to control the degree of spatial aggregation of the selected priority areas through the boundary length modifier parameter. We set this parameter to 0.001, based on the method proposed in Stewart and Possingham [50]. Another parameter which has to be calibrated when using MARXAN is the species penalty factor (the cost incurred if the representation target for a given species is not met, set for each species). We calibrated the species penalty factors according to an iterative method [51], gradually increasing the values until representation targets were met by the complete MARXAN solution in > 90% of the restarts. The numbers of restarts and iterations were set to 1,000 and 1,000,000, respectively.

For each species in each scenario we adjusted the representation target, so that the target would be achievable, i.e., calculated according to the remaining habitats that have not been lost to urban development over the 60 years.

We ran MARXAN separately for each land-cover scenario. For each run of MARXAN the input consists of the maps of potential habitat for all species under a specific land cover change scenario. For each scenario we used the same parameters and settings: Planning units (PUs) were 1 km^2^ hexagons arranged in a grid and clipped to the study region’s extent (a total of 8,257 PUs). For each scenario, PUs with > 50% built-up land cover or > 50% PAs were marked either as unavailable for selection or already protected, respectively.

### Construction and assessment of conservation priority area portfolios

We first constructed a conservation priority area portfolio based only on the present-day distributions of breeding bird habitats, by selecting the PUs with selection frequency higher than 90%. The total area of natural land cover in this portfolio was 2,131 km^2^. Hereinafter, we refer to this portfolio as the *current distributions portfolio*.

One of our objectives was to assess the tradeoffs that are associated with planning for only one of the possible scenarios and ignoring the other(s) (e.g., assuming that in the future, urban development will follow a regulated policy and ignoring a possible scenario of unregulated urban development). To this end, we constructed portfolios that were optimized separately for each of the values of the three selected variables affecting land-cover change processes: urban development policy, vegetation dynamics, and development rate. Table 1 shows the different portfolios and the scenarios that were assumed in each portfolio. For example, in order to construct a conservation portfolio optimized for a regulated urban development policy, we used the MARXAN results (selection frequencies) from the six scenarios that included regulated urban development.

There are different ways to combine the results of MARXAN runs for the different scenarios and thus to construct conservation area portfolios that are based on more than one MARXAN run. The approach we chose sought to identify the planning units that received high selection frequencies most consistently across the different scenarios. We therefore wanted to identify the highest-ranking planning units with the lowest variance across scenarios. To this end we performed the following steps: (1) calculated the coefficient of variation for each planning unit (ratio of standard deviation to mean selection frequency); (2) sorted the planning units in ascending order according to the coefficient of variation and then by descending order according to the mean; and (3) selected from among the highest ranking planning units as many as were needed to correspond to 2,131 km^2^ of natural land cover (same as the current distributions portfolio)–this step was taken in order to ensure that the portfolios we compared had the same area.

We evaluated the performance of a given portfolio by counting the number of species for which representation targets were met in each scenario and calculating the average and standard deviation of this value across the different scenarios for each portfolio. We compared portfolio performance under the scenarios that were used to construct a given portfolio, and portfolio performance under the alternative scenarios, and across all scenarios. Our objective was to identify the most robust portfolio, i.e., that which would be most immune to errors [52]. An error in this sense corresponds to a hypothetical case of planning for scenario A and achieving low performance if scenario B is realized. Thus, in our case, a robust portfolio is one that which consistently achieves a higher number of covered species, across the entire range of scenarios [18]–maximal return with minimal variance.

We performed a Binomial Generalized Linear model [53] using R [54] and the lme4 package [55]. This analysis served to examine whether there were significant differences in the number of covered species between the portfolios constructed using the different strategies. The binary response variable was whether the representation target was met for a given species in a given scenario under a given strategy. We entered the different strategies as fixed effects, and the species and scenarios as random effects (random intercept). We performed post-hoc pairwise comparisons of the different strategies using Tukey's HSD in the multcomp [56] and lsmeans [57] R packages. The data used to perform these statistical analyses in provided in S1–S3 Datasets.

## Results

**Fig 2:**
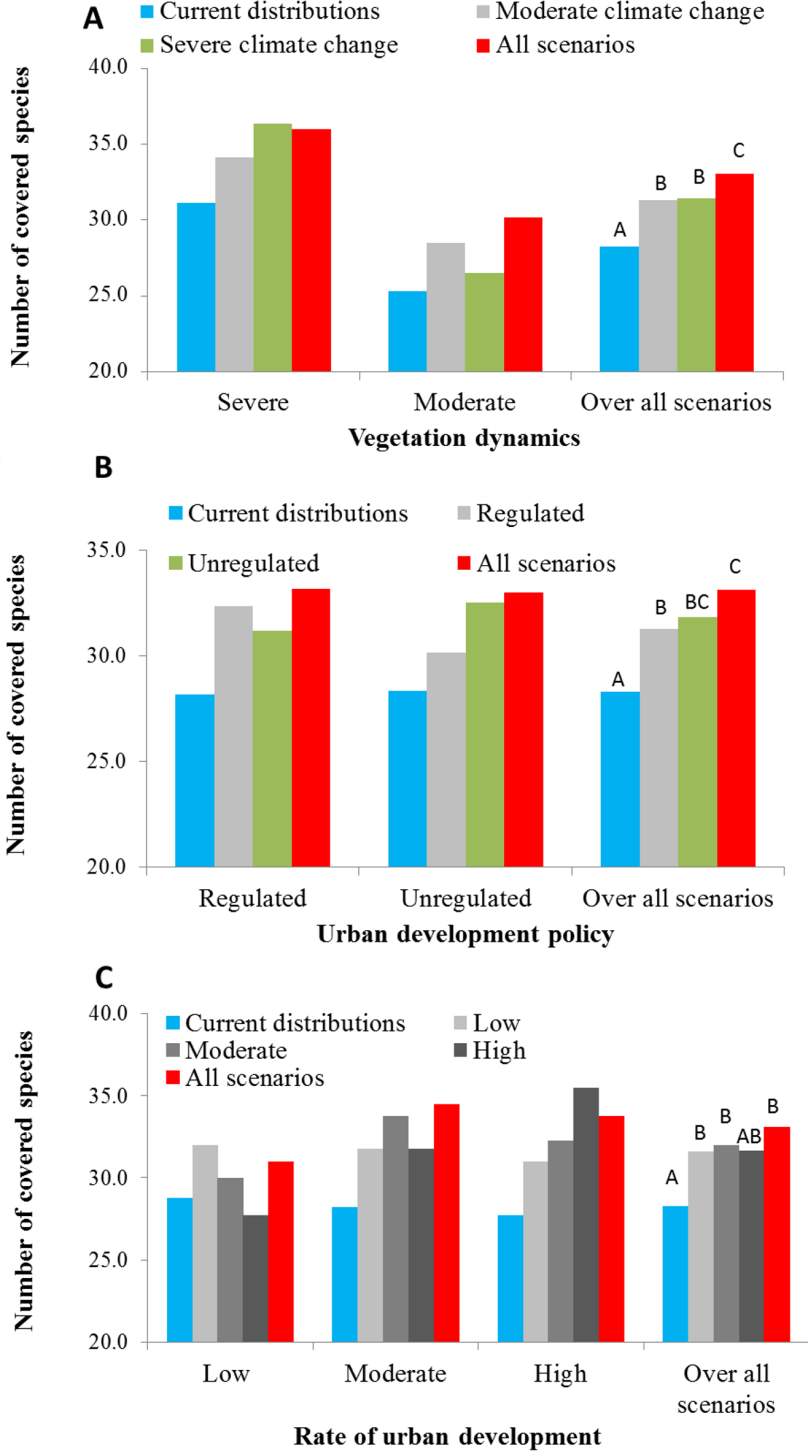
Comparison of portfolio performance. Performance (average number of covered species, i.e., species for which representation targets are met in a given scenario) of portfolios constructed based on (a) vegetation dynamics scenarios; (b). urban development policy scenarios; and (c)rate of development scenarios. The letters over the right series represent the results of the binary mixed-effect logistic regression model that compared the performance of the different portfolios.

Fig 2 presents graphically the comparison between the different portfolios. In most cases, the current distributions portfolio covers a smaller number of species compared to the other portfolios. The values on the Y-axis are the average number of species covered (representation target met) by portfolios (indicated by different column colors) across the group of scenarios (indicated in the X-axis categories). On average, the current distributions portfolios across all scenarios covers 28 species (out of a total of 48 species), whereas portfolios constructed based on a specific scenario cover an approximate average of 31 species, and the all-scenarios portfolio (based on all scenarios) covered approximately 33 species.

It is important to note that by definition the portfolios we compared do not cover all of the 48 study species. The reason for this is that we sought to compare portfolios of equal area (2,131 km^2^), and therefore when constructing the portfolios we selected a subset (the 90% most frequently selected planning units, see section 2.7) of the total area that would have been necessary to cover all of the species.

While these difference are relatively small, the results of the binomial generalized linear model comparing the performance of the portfolios constructed using the different strategies revealed significant differences in the performance (number of species covered, i.e., meeting representation target, under each portfolio). The detailed results of the binomial generalized linear models are provided in S1 Appendix.

The most consistent finding of these comparisons was that the performance of conservation priority areas based only on the current distributions (baseline portfolio) was significantly poorer than the other portfolios, and resulted in the lowest numbers of representation targets met (Fig 2, Tables 2–4). Another consistent result was that the all-scenarios portfolio covered a higher number of species than the other portfolios, although this result was not significant in all cases (Fig 2, Tables 2–4).

**Table 2:** P-values for Multiple Tukey's LSD pairwise comparison of portfolios for vegetation dynamics scenarios.

Portfolios	Current distributions	Severe climate change	Moderate climate change	All-scenarios
Current distributions		< 0.0001	< 0.0001	< 0.0001
Severe climate change			0.99	0.0471
Moderate climate change				0.0332
All-scenarios				

**Table 3:** P-values for Multiple Tukey's LSD Multiple pairwise comparison of portfolios for urban development policy.

Portfolios	Current distributions	Regulated development	Unregulated development	All-scenarios
Current distributions		< 0.0001	< 0.0001	< 0.0001
Regulated development			0.78	0.0197
Unregulated development				0.1928
All-scenarios				

**Table 4:** P-values for Multiple Tukey's LSD Multiple pairwise comparisons of portfolios for urban development rate.

Portfolios	Current distributions	Low development	Moderate development	High development	All-scenarios
Current distributions		< 0.0001	< 0.0001	0.79	< 0.0001
Low development			0.96	1.0	0.14
Moderate development				1.0	0.45
High development					0.99
All-scenarios					

Thirdly, differences in performance between portfolios that assumed only one of the alternative scenarios were insignificant. For example, across the examined scenarios, the severe and moderate climate change portfolios covered on average a similar number of species (31.3–31.4; Fig 2C).

For portfolios constructed based on vegetation dynamics scenarios, the number of species covered by portfolio based on all scenarios was significantly higher than all the other portfolios (Table 2, Fig 2A), and the number of species covered by the baseline portfolio was significantly lower than all the other portfolios (Table 2, Fig 2A).

For portfolios constructed based on scenarios of different urban development policies, the number of species covered under the baseline portfolio was significantly lower compared to all other portfolios (Table 3, Fig 2B). The all-scenarios portfolio covered a significantly higher number of species compared to the regulated development portfolio and the baseline portfolio.

Also for portfolios constructed based on scenarios of different urban development rates, the number of species covered under the baseline portfolio was significantly lower compared to all other portfolios, except in comparison to the high development rate portfolio (Table 4, Fig 2C). In this case, the all-scenarios portfolio did not cover a significantly higher compared to any of the scenarios based on a single development rate.

**Fig 3:**
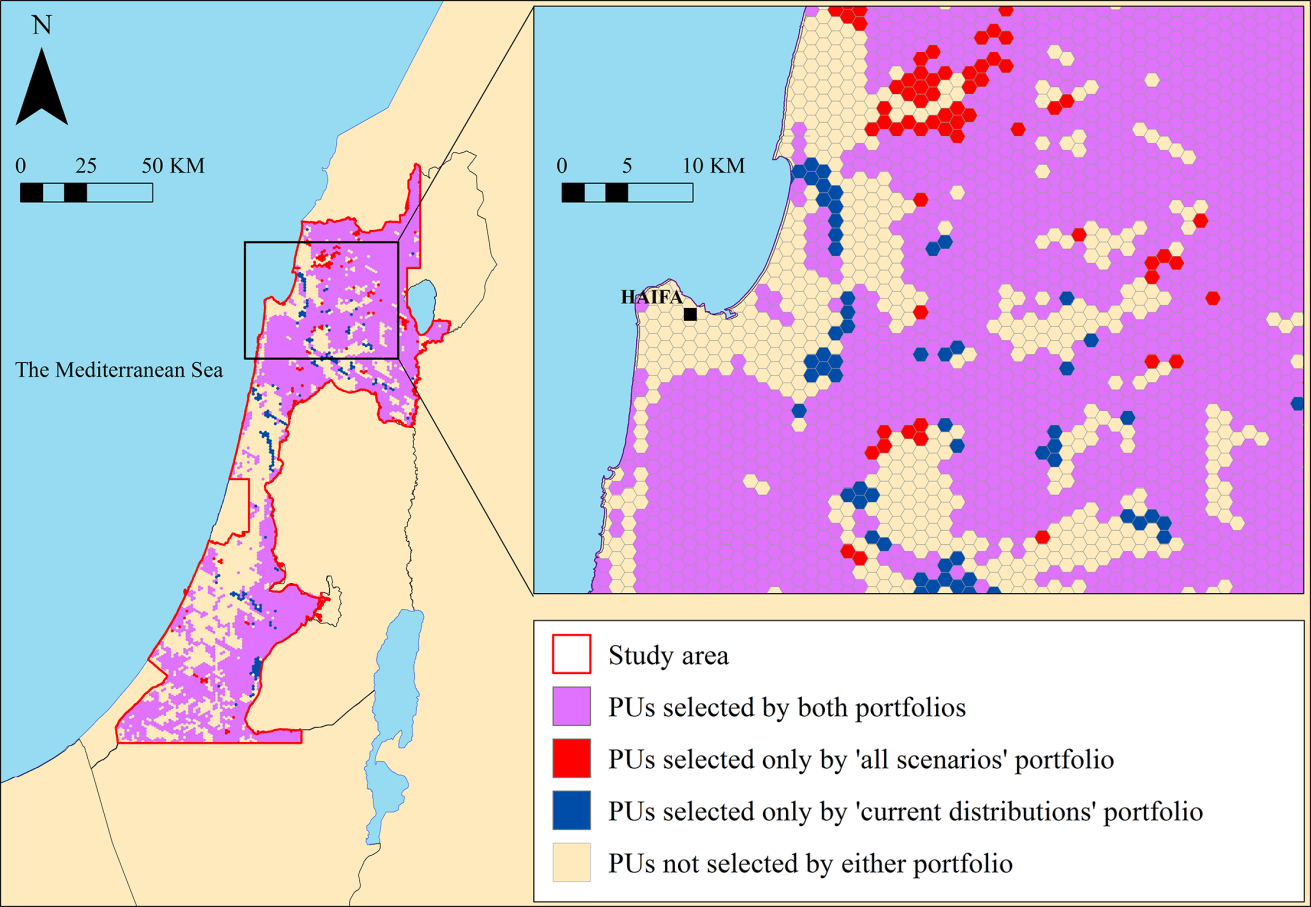
Spatial comparison of the all-scenarios and current distributions portfolios.

Table 5 shows the percentage of overlapping areas between the different portfolios, i.e., when two alternative portfolios are compared, how much of their area overlaps and how much area is unique to a specific portfolio (see Fig 3 for an example). In all cases, the degree of spatial overlap between the portfolios was above 90% (values above diagonal in Table 5). The overlap between the land-cover classes in the scenarios used to construct the portfolios was lower, ranging between 32% and 76% (values below diagonal in Table 5). For example, the spatial overlap between the moderate and severe climate change portfolios was 96% while the overlap between vegetation classes in these two scenarios was approximately 59%.

**Table 5:** Percentage of overlap between portfolios.

	Current distributions	All-scenarios	Severe climate change	Moderate climate change	Regulated Development	Unregulated Development	Low development	Moderate development	High development
Current distributions	-	94.1	93.2	93.4	94.0	94.1	94.5	94.4	93.2
All-scenarios		-	97.2	97.9	98.7	98.8	97.5	99.2	97.3
Severe climate change	38.2		-	96.0					
Moderate climate change	45.1		58.5	-					
Regulated Development	53.8				-	97.6			
Unregulated Development	46.9				76.0	-			
Low Development	73.5						-	97.2	95.1
Moderate Development	45.7						75.0	-	96.9
High Development	32.0						69.4	74.6	-

Above diagonal: A cell-by-cell comparison between the entire portfolios (i.e., all land cover classes within the selected planning units); Below diagonal: a comparison of overlap between specific land-cover classes in the different portfolios. For the portfolios constructed for vegetation dynamics scenarios, we compared the average overlap between the five different classes of Mediterranean vegetation. For the comparison between the portfolios constructed for urban development (policy and rate of development), we compared the overlap between built-up land class.

## Discussion

Our study joins several systematic conservation planning studies that have incorporated future uncertainty in both ecological processes and processes considered threats to biodiversity. We constructed and modeled future scenarios for two important land-cover change processes: vegetation succession and disturbance (habitat dynamics, an ecological process) and urban development (a form of habitat loss, a threat to biodiversity). We constructed conservation area portfolios using several strategies and analyzed their performance across the range of future scenarios. Our analyses revealed several main findings.

First, the current distributions portfolio (conservation areas based only on the current distributions of breeding bird habitats) did not perform as well as portfolios that incorporated information from future scenarios, covering on average between 3 to 5 species less–this result was significant and consistent for nearly all cases (except for the difference between the current distributions portfolio and the high development rate portfolio which was not significant) (Fig 2, Tables 2–4): On average the current distributions portfolio covered 28 species, whereas portfolios based on specific scenarios and on all scenarios covered an average of approximately 31 and 33 species, respectively. Veloz et al. [22] obtained similar results: they found that selection of priority areas based only on current distributions had the poorest performance when compared to strategies (portfolios) that incorporated future scenarios [22]. This result is expected, since we included extreme scenarios, to ensure that the array of scenarios cover the range of most possible scenarios, including some that are not very likely. Indeed, this result provides strong support for the incorporation of scenario planning in systematic conservation planning.

A second main finding is that the all-scenarios portfolio (inclusion of all possible scenarios in the conservation area portfolio) was the most robust portfolio. The all-scenarios portfolio consistently outperformed scenario-specific portfolios in case of error (occurrence of alternative scenario). This result was consistent and significant in some of cases–the differences in favor of the all-scenarios portfolio were significant when compared to both vegetation dynamics portfolios (Table 2, Fig 2A) and to the regulated urban development policy (Table 3, Fig 2B). Overall however, the differences in the number of species covered by the all-scenarios portfolio compared to scenario specific portfolios were relatively small–approximately 2 species. One implication of this result is that relying on a worst-case scenario approach is not necessarily a robust approach and even a risky strategy. For example, the portfolio based on a worst case scenario of severe climate change was outperformed by the all-scenario portfolio. Examining Fig 2A, it can be seen that if the severe climate change scenario is realized the performance of the all-scenarios portfolio and the severe climate change portfolio would be relatively similar: covering approximately 36 species (middle X-axis category). However, if the moderate climate change scenario is realized, the severe climate change portfolio would result in the coverage of only 26.5 species on average compared with 30.2 under the all-scenarios portfolio (left-most X-axis category). Assuming a worst-case scenario is a common approach in environmental impact, climate change and conservation planning studies [33,58,59]. However, this type of approach could be insufficient or lead to inadequate spread of resources (e.g., selection of conservation areas) [10,18]. Our results echo findings of other studies, which demonstrated that the strategy of including all scenarios (assigning equal weights to future scenarios and selecting the common priority areas) yielded the most robust performance under future uncertainty. Carvalho et al. [18] found that investing beforehand in a worst-case scenario was not the most efficient approach and that the areas selected consistently in all scenarios offered the least investment risk [18]. Veloz et al. [22] found that a strategy incorporating all possible strategies was more robust than strategies that relied on a single possible scenario, given the possibility of "error," i.e., making an incorrect prediction in terms of the selected scenario [22].

We constructed the portfolios so that the total area of natural land cover would be identical. In general, the spatial differences between the different portfolios (overlap higher than 90% in most cases; Table 5) were small relative to those found in other studies [8,20]. We evaluated a broad range of possible future trajectories and included some extreme scenarios (e.g., the high development rate scenarios), which are considered by some as highly unlikely, and assigned to these scenarios the same weights as we assigned to scenarios that are often considered more likely. However, given that this strategy accounts for a broad range of possible scenarios, it is feasible that the majority of conservation priority areas identified would be similar. Nevertheless, we believe that the large degree of overlap between the different portfolios is an idiosyncratic result of our case study. Our study area is dominated by built-up areas and agricultural land (Table B in S1 Supporting information). The relative proportion of Mediterranean vegetation classes, which are the most important habitats for the study species, is relatively small. Thus, most of the highly valuable areas of Mediterranean vegetation are consistently selected in all portfolios.

From a practical perspective, this result suggests that most of the priority areas identified by current bird species distributions are expected to retain their importance in the future. Similar results have been found in other studies [18,60,61]. In a previous study we demonstrated that the study region's current protected area system does not provide adequate protection to the existing available breeding bird habitats: representation targets are met for only 23% of the study species [40] and recommended that additional areas be protected (potentially according to the same current distributions portfolio discussed and analyzed in the present study). Given the large degree of overlap between the different portfolios that we examined (current distributions portfolio, portfolios based on specific scenarios, and the all-scenarios portfolio), our main policy recommendation would be to concentrate on the conservation of the areas that overlap between all of the portfolios (e.g., pink colored areas in Fig 3). If this is achieved, further preparation for the range of scenarios we examined can be achieved by adding relatively few areas. It should be noted that in order to provide recommendations that are more relevant to the actual situation, a similar analysis should be conducted using a more realistic land cost model. In the present study the cost of each parcel was equal, and our portfolios minimized the area necessary for conservation. However the actual situation is that the cost of parcels (planning units) varies and depends on factors such as proximity to major cities, their statutory planning status and their suitability for other uses such as development or agriculture.

## Conclusions

We proposed and demonstrated an approach to systematic conservation planning that incorporates both anthropogenic threats and ecological processes, taking into account the uncertainty regarding their outcomes, and enables a comparison of various strategies for allocating conservation resources. The fact that planning based only on the current conditions performed more poorly under future scenarios is not surprising; however, a large number of conservation planning studies still rely only on present or past distributions of biodiversity features. Our findings provide further support for the use of scenario planning in systematic conservation planning. Our most important conclusion is that compared to the strategies that assume a worst-case scenario or assign a high degree of likelihood to a specific scenario, the strategy that provided the highest degree of robustness to future uncertainty was that which takes into account the entire range of plausible scenarios when selecting areas for conservation. This result calls attention to the importance of constructing a meaningful set of scenarios and considering the entire plausible range of future alternatives, rather than assuming the certainty of specific scenarios or relying on the robustness of the worst-case scenario approach.

## Supporting information

S1 Appendix Results of binomial generalized linear models comparing performance of portfolios. (DOCX)

S1 Dataset Dataset used for binomial generalized linear model comparing performance of portfolios under different vegetation dynamics scenarios. (XLSX)

S2 Dataset Dataset used for binomial generalized linear model comparing performance of portfolios under different scenarios of urban development policies. (XLSX)

S3 Dataset Dataset used for binomial generalized linear model comparing performance of portfolios under different scenarios of urban development rate. (XLSX)

S1 Table The target species and their habitat-associations. (DOCX)

S1 Supporting information Land-cover data sources and percentages of land-cover classes in study area. (DOCX)

S2 Supporting information Description and methods of land-cover simulation model. (DOCX)
