# Supplementary material for: Conservation planning under uncertainty in urban development and vegetation dynamics
Source: PLoS One. 2018 Apr 5;13(4):e0195429. doi: 10.1371/journal.pone.0195429 (PMC5886564; doi:10.1371/journal.pone.0195429)
Supplement: S1 Appendix — (DOCX) [file pone.0195429.s001.docx]

**S6 Appendix - Results of binomial generalized linear models**

1. **Model comparing performance of portfolios under different vegetation dynamics scenarios**

Table 1A: Random effects

| **Groups** | **Variance** | **Std.Dev.** |
| --- | --- | --- |
| species (intercept) | 270.70 | 16.453 |
| Scenario (intercept) | 2.23 | 1.493 |

Number of obs: 2352, groups: species, 48; scenario, 12

Table 1B: Fixed effects

|  | **Estimate** | **Std.**  **Error** | **z value** | **Pr(>\|z\|)** |
| --- | --- | --- | --- | --- |
| **Intercept** | 12.70238 | 1.36125 | 9.331 | < 2e-16 |
| **Moderate climate change** | -0.03457 | 0.26295 | -0.131 | 0.89540 |
| **Current distributions** | -1.39713 | 0.28240 | -4.947 | 7.52e-07 |
| **All scenarios** | 0.68480 | 0.26428 | 2.591 | 0.00956 |

Table 1C: Correlation of Fixed Effects:

|  | **Intercept** | **Moderate climate change** | **Current**  **distributions** |
| --- | --- | --- | --- |
| **Moderate**  **climate change** | -0.098 |  |  |
| **Current**  **distributions** | -0.202 | 0.467 |  |
| **All scenarios** | -0.060 | 0.496 | 0.434 |

Table 1D: Simultaneous Tests for General Linear Hypotheses - Multiple Comparisons of Means: Tukey Contrasts

|  | Estimate | Std. Error | z value | Pr(>\|z\|) |
| --- | --- | --- | --- | --- |
| Moderate climate change –  severe climate change | -0.03457 | 0.26295 | -0.131 | 0.9992 |
| Current distributions –  severe climate change | -1.39713 | 0.28240 | -4.947 | <0.001 |
| All scenarios –  severe climate change | 0.68480 | 0.26428 | 2.591 | 0.0471 |
| Current distributions –  moderate climate change | -1.36256 | 0.28212 | -4.830 | <0.001 |
| All scenarios –  moderate climate change | 0.71937 | 0.26461 | 2.719 | 0.0331 |
| All scenarios –  current distributions | 2.08193 | 0.29127 | 7.148 | <0.001 |

1. **Model comparing performance of portfolios under different scenarios of urban development policy.**

Table 2A: Random effects

| **Groups** | **Variance** | **Std.Dev.** |
| --- | --- | --- |
| species (intercept) | 264.084 | 16.251 |
| Scenario (intercept) | 1.873 | 1.369 |

Number of obs: 2352, groups: species, 48; scenario, 12

Table 2B: Fixed effects

|  | **Estimate** | **Std. Error** | **z value** | **Pr(>\|z\|)** |
| --- | --- | --- | --- | --- |
| **Intercept** | 11.0733 | 1.3374 | 8.280 | < 2e-16 |
| **Unregulated urban development** | 1.6194 | 0.2895 | 5.594 | 2.22e-08 |
| **Regulated urban**  **development** | 1.3693 | 0.2869 | 4.773 | 1.81e-06 |
| **All scenarios** | 2.1522 | 0.2976 | 7.231 | 4.79e-13 |

Table 2C: Correlation of Fixed Effects:

|  | **Intercept** | **Unregulated**  **urban development** | **Regulated urban development** |
| --- | --- | --- | --- |
| **Unregulated**  **urban development** | 0.005 |  |  |
| **Regulated**  **urban development** | -0.006 | 0.569 |  |
| **All scenarios** | 0.024 | 0.583 | 0.573 |

Table 2D: Simultaneous Tests for General Linear Hypotheses Multiple Comparisons of Means: Tukey Contrasts

|  | Estimate | Std. Error | z value | Pr(>\|z\|) |
| --- | --- | --- | --- | --- |
| Unregulated  urban development – current  distributions | 1.6194 | 0.2895 | 5.594 | <0.001 |
| Regulated urban  development –  current distributions | 1.3693 | 0.2869 | 4.773 | <0.001 |
| All scenarios –  current distributions | 2.1522 | 0.2976 | 7.231 | <0.001 |
| Regulated urban  development –  unregulated  urban development | -0.2501 | 0.2677 | -0.934 | 0.7860 |
| All scenarios –  unregulated urban  development | 0.5328 | 0.2681 | 1.987 | 0.1924 |
| All scenarios –  regulated urban  development | 0.7829 | 0.2702 | 2.897 | 0.0198 |

1. **Model comparing performance of portfolios under different scenarios of urban development rate.**

Table 3A: Random effects

| **Groups** | **Variance** | **Std.Dev.** |
| --- | --- | --- |
| species (intercept) | 253.912 | 15.935 |
| Scenario (intercept) | 1.442 | 1.201 |

Number of obs: 2928, groups: species, 48; scenario, 13

Table 3B: Fixed effects

|  | **Estimate** | **Std. Error** | **z value** | **Pr(>\|z\|)** |
| --- | --- | --- | --- | --- |
| **Intercept** | 12.36299 | 1.78694 | 6.919 | 4.56e-12 |
| **Low**  **development rate** | -0.02991 | 1.27222 | -0.024 | 0.981 |
| **Moderate**  **development rate** | 0.13510 | 1.27221 | 0.106 | 0.915 |
| **Current**  **distributions** | -1.42876 | 1.27563 | -1.120 | 0.263 |
| **All scenarios** | 0.56367 | 1.27258 | 0.443 | 0.658 |

Table 3C: Correlation of Fixed Effects

|  | **Intercept** | **Low**  **development**  **rate** | **Moderate**  **development**  **rate** | **Current**  **distributions** |
| --- | --- | --- | --- | --- |
| **Low**  **development rate** | -0.646 |  |  |  |
| **Moderate**  **development rate** | -0.645 | 0.980 |  |  |
| **Current**  **distributions** | -0.660 | 0.977 | 0.977 |  |
| **All scenarios** | -0.642 | 0.979 | 0.980 | 0.976 |

Table 3D: Simultaneous Tests for General Linear Hypotheses - Multiple Comparisons of Means: Tukey Contrasts

|  | Estimate | Std. Error | z value | Pr(>\|z\|) |
| --- | --- | --- | --- | --- |
| Low development –  high development | -0.02991 | 1.27222 | -0.024 | 1.000 |
| Moderate development –  high development | 0.13510 | 1.27221 | 0.106 | 1.000 |
| Current distributions –  high development | -1.42876 | 1.27563 | -1.120 | 0.772 |
| All scenarios –  high development | 0.56367 | 1.27258 | 0.443 | 0.991 |
| Moderate development –  low development | 0.16501 | 0.25704 | 0.642 | 0.963 |
| Current distributions –  low development | -1.39885 | 0.27416 | -5.102 | <0.001 |
| All scenarios -  low development | 0.59358 | 0.25846 | 2.297 | 0.126 |
| Current distributions –  moderate development | -1.56386 | 0.27551 | -5.676 | <0.001 |
| All scenarios –  moderate development | 0.42857 | 0.25763 | 1.663 | 0.421 |
| All scenarios –  current distributions | 1.99243 | 0.28049 | 7.103 | <0.001 |
