# Supplementary material for: Conservation planning under uncertainty in urban development and vegetation dynamics
Source: PLoS One. 2018 Apr 5;13(4):e0195429. doi: 10.1371/journal.pone.0195429 (PMC5886564; doi:10.1371/journal.pone.0195429)
Supplement: S1 Table — (DOCX) [file pone.0195429.s005.docx]

Conservation planning under uncertainty in urban development and vegetation dynamics

David Troupin^*^ and Yohay Carmel

* Corresponding author: Faculty of Architecture and Town Planning, Technion – Israel Institute of Technology, Haifa 32000, Israel. Tel.: 972-54-7910799. Email: [davidtroupin@gmail.com](mailto:davidtroupin@gmail.com)

**S1** – Table listing the target species and their habitat-associations. Regional IUCN status is based on the Red Book of Vertebrates in Israel (Dolev and Perevolotsky, 2002).

| **No.** | **Scientific name** | **Moderate/strong habitat association with** | | | | | | | | **Regional IUCN status** | |
| --- | --- | --- | --- | --- | --- | --- | --- | --- | --- | --- | --- |
|  |  | **Herbaceous vegetation** | **Sparse shrublands** | **Dense shrublands** | **Sparse woodlands** | **Dense woodlands** | **Planted forests** | **Riparian vegetation** | **Cliffs** | |  |
| 1 | *Accipiter nisus* |  |  |  | V | V | V |  |  | | LC |
| 2 | *Alectoris chukar* | V | V |  | V |  |  |  |  | | NT |
| 3 | *Anthus similis* | V | V | V |  |  |  |  | V | | EN |
| 4 | *Asio otus* |  |  |  | V |  | V |  |  | | LC |
| 5 | *Athene noctua* | V | V |  |  |  |  |  | V | | NT |
| 6 | *Bubo bubo* | V | V |  | V |  | V |  | V | | NT |
| 7 | *Burhinus oedicnemus* | V | V |  | V |  | V |  |  | | NT |
| 8 | *Buteo rufinus* | V | V |  | V |  |  |  | V | | NT |
| 9 | *Calandrella brachydactyla* | V |  |  |  |  |  |  |  | | DD |
| 10 | *Carduelis cannabina* |  | V | V | V |  | V |  |  | | NT |
| 11 | *Carduelis carduelis* | V | V | V | V |  | V |  |  | | NT |
| 12 | *Carduelis chloris* | V | V | V | V |  | V |  |  | | LC |
| 13 | *Cercotrichas galactotes* |  | V | V | V |  | V | V |  | | NT |
| 14 | *Cercomela melanura* | V |  |  |  |  |  |  | V | | LC |
| 15 | *Circaetus gallicus* | V | V | V | V | V | V |  | V | | LC |
| 16 | *Coracias garrulus* |  | V |  | V |  |  |  | V | | NT |
| 17 | *Corvus monedula* | V |  |  | V |  | V |  | V | | LC |
| 18 | *Cuculus canorus* | V | V | V | V |  |  | V |  | | NT |
| 19 | *Emberiza caesia* |  | V | V | V |  | V |  |  | | NT |
| 20 | *Emberiza melanocephala* |  | V | V | V | V |  |  |  | | VU |
| 21 | *Falco subbuteo* |  |  |  | V |  | V |  |  | | NT |
| 22 | *Francolinus francolinus* |  | V |  |  |  |  |  |  | | VU |
| 23 | *Galerida cristata* | V | V | V | V |  | V |  |  | | NT |
| 24 | *Gyps fulvus* | V | V |  |  |  |  |  | V | | VU |
| 25 | *Hippolais languida* |  | V | V | V |  |  |  |  | | NT |
| 26 | *Hippolais olivetorum* |  |  |  | V | V | V |  |  | | DD |
| 27 | *Hippolais pallida* |  | V | V | V | V | V | V |  | | LC |
| 28 | *Lanius collurio* |  | V | V | V |  |  |  |  | | NT |
| 29 | *Lanius excubitor* | V | V | V | V |  |  |  |  | | LC |
| 30 | *Lanius nubicus* |  | V |  | V |  | V |  |  | | NT |
| 31 | *Lanius senator* | V | V | V | V |  | V |  |  | | NT |
| 32 | *Melanocorypha calandra* | V |  |  |  |  |  |  |  | | NT |
| 33 | *Merops apiaster* |  |  |  | V |  | V |  |  | | VU |
| 34 | *Miliaria calandra* | V | V | V | V |  | V |  |  | | LC |
| 35 | *Muscicapa striata* |  |  |  | V |  | V |  |  | | LC |
| 36 | *Oenanthe hispanica* | V | V |  | V |  | V |  |  | | LC |
| 37 | *Oenanthe isabellina* | V | V |  |  |  |  |  |  | | LC |
| 38 | *Oriolus oriolus* |  |  |  | V |  | V |  |  | | NT |
| 39 | *Passer hispaniolensis* | V | V |  | V |  |  | V |  | | LC |
| 40 | *Petronia petronia* |  | V |  |  |  |  |  | V | | LC |
| 41 | *Rhodopechys obsoleta* |  | V |  | V |  | V |  |  | | LC |
| 42 | *Scotocerca inquieta* |  | V |  |  |  |  |  |  | | NT |
| 43 | *Sylvia communis* | V | V | V | V |  |  |  |  | | LC |
| 44 | *Sylvia conspicillata* |  | V | V |  |  |  |  |  | | VU |
| 45 | *Sylvia curruca* |  | V | V | V | V | V | V |  | | LC |
| 46 | *Sylvia hortensis* |  | V | V | V | V |  |  |  | | DD |
| 47 | *Sylvia melanocephala* |  | V | V | V | V | V | V |  | | DD |
| 48 | *Troglodytes troglodytes* |  | V | V | V | V | V |  |  | | LC |
