# Supplementary material for: Conservation planning under uncertainty in urban development and vegetation dynamics
Source: PLoS One. 2018 Apr 5;13(4):e0195429. doi: 10.1371/journal.pone.0195429 (PMC5886564; doi:10.1371/journal.pone.0195429)
Supplement: S1 Supporting information — (DOCX) [file pone.0195429.s006.docx]

Conservation planning under uncertainty in urban development and vegetation dynamics

David Troupin^*^ and Yohay Carmel

* Corresponding author: Faculty of Architecture and Town Planning, Technion – Israel Institute of Technology, Haifa 32000, Israel. Tel.: 972-54-7910799. Email: [davidtroupin@gmail.com](mailto:davidtroupin@gmail.com)

**S2 Supporting Information –** **Land-cover data sources and percentages** **of land-cover classes in study area**.

**Table A.** Land-cover data sources.

| **Data** | **Source** | **Year** |
| --- | --- | --- |
| LC/vegetation | Israel Nature and Parks Authority | 1995 |
| Agricultural plantations and croplands | Israel Central Bureau of Statistics | 2002 |
| Built-up areas^[[1]](#footnote-1)^ | Israel Ministry of Interior | 2007 |
| Cliffs, Running streams, Water bodies | The Hebrew University GIS center | 2008 |
| LC/Vegetation in JNF managed areas | Jewish National Fund | 2009 |

**Table B.** Land-cover classes in the entire study.

| **No.** | **Class** | **% of study area** | |
| --- | --- | --- | --- |
| 1 | Herbaceous vegetation | | 2.13 |
| 2 | Sparse shrubs | | 3.06 |
| 3 | Dense shrubs | | 2.39 |
| 4 | Sparse trees | | 5.39 |
| 5 | Dense trees | | 7.42 |
| 6 | Planted forest | | 9.25 |
| 7 | Other natural land cover | | 2.76 |
| 8 | Riparian vegetation | | 0.52 |
| 9 | Agricultural plantations (orchards, groves, etc.) | | 14.60 |
| 10 | Croplands | | 38.00 |
| 11 | Water bodies (fish ponds, water reservoirs, etc.) | | 1.08 |
| 12 | Built-up areas | | 13.40 |
| 13 | Cliffs | | 0.01 |
|  | Total area (km^2^) | | 7,804 |

1. Built-up areas include man-made development such as structures and buildings etc. They do not include infrastructure such as roads or powerlines. [↑](#footnote-ref-1)
