# Supplementary material for: Conservation planning under uncertainty in urban development and vegetation dynamics
Source: PLoS One. 2018 Apr 5;13(4):e0195429. doi: 10.1371/journal.pone.0195429 (PMC5886564; doi:10.1371/journal.pone.0195429)
Supplement: S2 Supporting information — (DOCX) [file pone.0195429.s007.docx]

Conservation planning under uncertainty in urban development and vegetation dynamics

David Troupin^*^ and Yohay Carmel

* Corresponding author: Faculty of Architecture and Town Planning, Technion – Israel Institute of Technology, Haifa 32000, Israel. Tel.: 972-54-7910799. Email: [davidtroupin@gmail.com](mailto:davidtroupin@gmail.com)

**Appendix 2 -** **Description and methods of land-cover simulation model**

# **Simulation framework**

We implemented the simulation of urban development and vegetation dynamics using DINAMICA-EGO [1], a cellular-automata based simulation model. It has two transition allocation functions that operate on the basis of local CA rules: “*patcher*” and “*expander*”. The size of new patches and expansion areas is set according to a lognormal probability distribution. The user specifies the mean size and variance of each type of patch/expansion area to be formed. The “*patcher*” generates new patches of a certain class and the “*expander*” function serves to expand existing patches.

## **Vegetation dynamics scenarios**

### **Parameterization**

The different vegetation formations and transition pathways considered in the simulation are shown in Fig. 1.


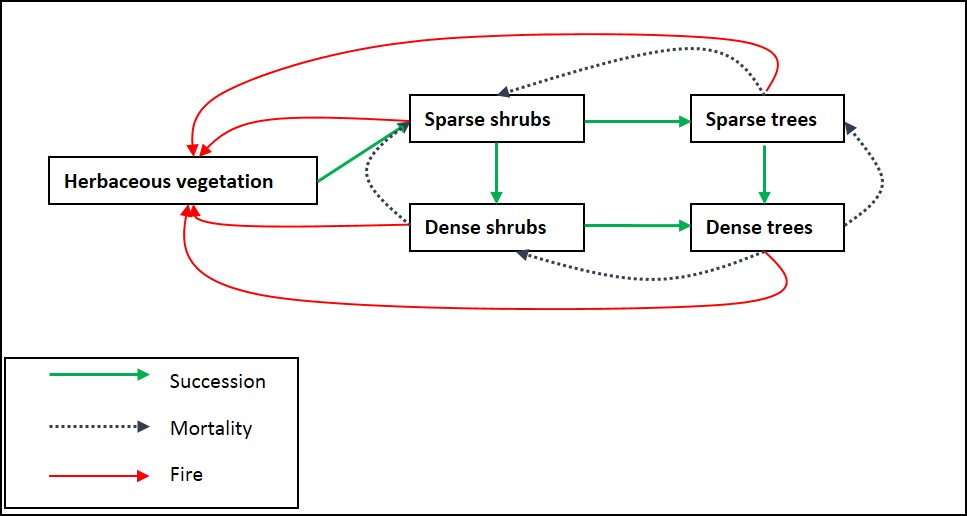


Fig. 1 The modeled transition pathways between the Mediterranean vegetation formations.

In order to determine the transition probabilities between the different vegetation formations, we relied on two studies that were conducted in different parts of the study area [2,3]. These two sites represent the extremes of precipitation levels within the study area (mean annual precipitation of 900 mm and 450 mm at Mount Meron and Adulam, respectively). The results of both studies (proportions of each vegetation formation at different time intervals) enabled the calculation of transition probabilities between the states of herbaceous vegetation, shrubs, and trees. The determination of the transition matrices for both scenarios and assignment transition matrices across the study area required several assumptions:

1. At the regional scale, in which the study is conducted, the variation across space in transition probabilities is determined by precipitation rates. Clearly transition probabilities could be modeled at a higher spatial resolution, and are affected by additional factors. However this type of modeling was beyond the scope of the study. Based on this assumption, we divided it into sub-regions according to three levels of mean annual precipitation: (a) low: <= 500 mm; (b) Intermediate: 550-650 mm; and (c) high: >= 700 mm (Fig. 2).


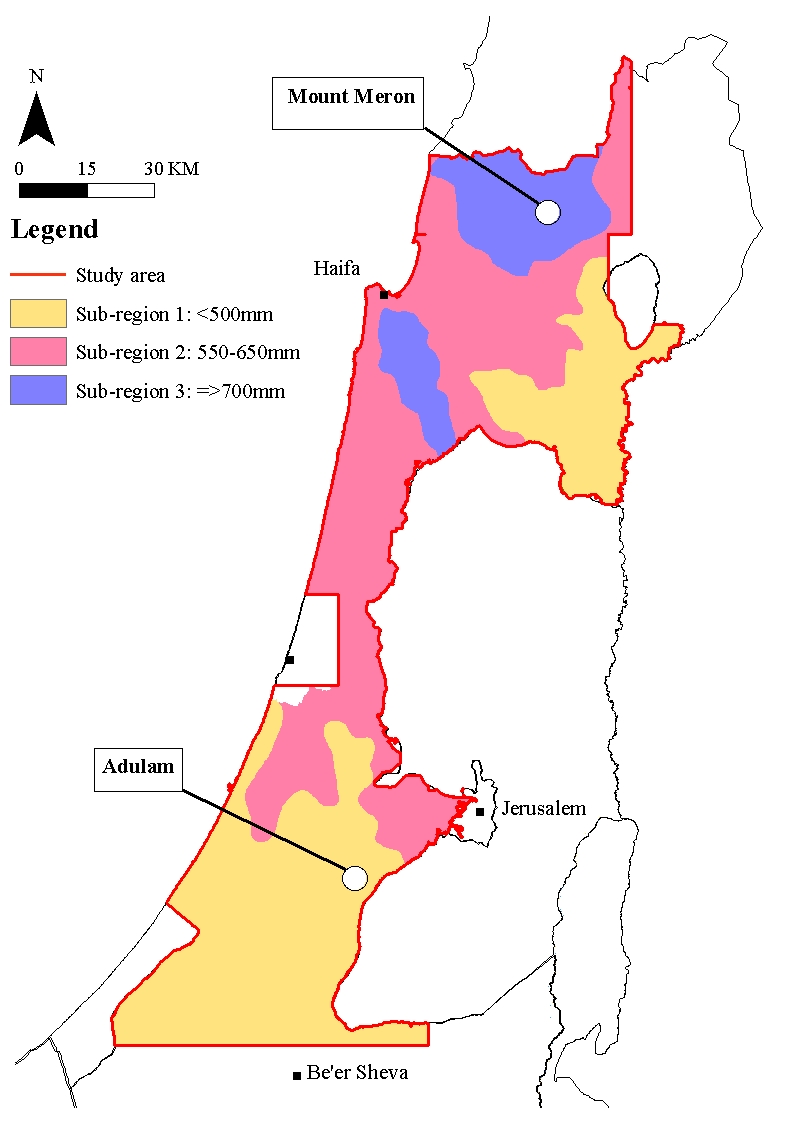


Fig. 2 Precipitation-based sub-regions used for simulations of vegetation dynamics (data provided by the Hebrew University GIS Center).

1. The studies we relied on did not include transition probabilities between different densities within a certain vegetation formation: i.e., sparse shrubs into dense shrubs, and sparse trees into dense trees. We therefore relied on expert opinion (Y. Carmel) and set the values for these two types of transition by reducing the transition probability of shrubs into trees found in the studies by 50%. In addition, a one-step transition from sparse shrubs into dense trees was not considered a possible pathway, as this type of change does not occur generally. This type of change may occur in two steps – first a transition from sparse shrubs into sparse trees or dense shrubs and then into dense trees (Y. Carmel).
2. For the moderate climate change scenario we used the values obtained from the two aforementioned studies. Thus, sub-regions of categories 1 and 3 were assigned the transition matrices corresponding to Adulam and Mount Meron, respectively. For sub-regions with an intermediate level of precipitation (category 2) we interpolated a transition matrix by averaging the values of categories 1 and 3.
3. Under the moderate climate change scenario, fire events were assumed to be the only cause of backward transitions (e.g., from trees to shrubs and herbaceous vegetation, and from shrubs to herbaceous vegetation). These backward transitions were set to a very small value (P=0.001). This assumption is consistent with the studies cited above as well as other vegetation dynamics studies conducted in the study area, e.g., [4].
4. For the severe climate change scenario, we shifted the transition matrices between the sub-regions, and assumed lower forward transition probabilities for the driest sub-region. In addition, in order to represent a decrease in shrub and tree cover we incorporated in the transition matrices for this scenario: (a) reduced forward transition rates (herbaceous vegetation into shrubs, and shrubs into trees) – representing decreased recruitment of woody species; and (b) increased backward transitions (shrubs to herbaceous vegetation and trees into herbaceous vegetation) – representing mortality of woody species and their replacement by herbaceous vegetation.

We incorporated fires as random events that result in the conversion of all types of shrub and tree formations into herbaceous vegetation. According to [5], the average annual area of woodland and shrubland that are burned and require restoration, is 8.4 km^2^. We used the fire record (number of events each year and the area of each event) for the years 1988-2006 provided by the Jewish National Fund in order to simulate fire events in the model. The fire record data does not enable differentiation between the severity of fire events or the type of vegetation burned. We therefore defined a minimal threshold of area burned (0.2 km^2^). From each year in the fire record we randomly selected fire events that met this area threshold until the total area of fire events reached the annual average of 8.4 km^2^ (for the moderate climate change scenario) and 16.8 km^2^ (to represent increased frequency of fire events for the severe climate change scenario). For each year in the simulation we then randomly selected and assigned one of these tables.

For each individual fire event, a random patch was selected out of all the shrub/tree patches equal in area or larger. A square grid comprised of cells (quadrants) with a size equal to the individual fire event was then placed over the randomly selected patch. Quadrants (individual cells from the square grid) that partially covered the random patch and were smaller than 90% of the individual fire event were eliminated. One of the remaining quadrants (with an area of at least 90% of the fire event) was then selected. If all the quadrants partially covered the patch and were smaller than 90% of the individual fire event, the entire patch was designated for that fire event.

### **Scenarios**

We constructed two scenarios of vegetation dynamics, corresponding to two contrasting emissions scenarios [6,7]:

#### **Scenario 1: Extreme climate change**

This scenario corresponds to the A2 emission scenario (extreme warming, following high greenhouse gas emissions). In this scenario, altered succession patterns and fire regime can be expected. Drier climate and altered rainfall regime [8,9] would result in lower recruitment and increased mortality of woody species (drought-induced mortality of both shrubs and trees) and in an increase in fire frequency – more frequent events and events of larger magnitude (leading to increased transition of woody vegetation into herbaceous vegetation).

#### **Scenario 2: Moderate climate change**

This scenario corresponds to the B2 emissions scenario (moderate warming following medium-low greenhouse gas emissions scenario). We assumed that under a moderate warming scenario, the patterns of succession and disturbance will be relatively similar to those that have been observed in the past decades. Woody vegetation cover is expected to gradually increase and the fire frequency is expected to remain unchanged.

## **Urban development**

### **Parameterization**

We used maps of built-up^[[1]](#footnote-1)^ areas from the years 1998, 2003 and 2007 provided by the Ministry of Interior to calculate the annual transition probabilities of non-built land into built-up land in each of the five administrative districts for the periods 1998-2003 and 2003-2007 using DINAMICA-EGO [1]. We used the built-up area layers and additional layers to calculate variables that would serve as drivers of spatial change (Table 1). We converted all vector GIS layers into raster format of 50 x 50m. We selected the variables for the spatial probability map based on previous studies in the study area that have either reported statistical association between these factors and development or related them to development through policy. We then used the Weight of Evidence method [10], a Bayesian method that calculates empirical relationships of spatial variables with respect to land cover changes [1], to create a map of spatial transition probability.

Table 1 Variables used as drivers of open-built transition in the spatial probability map listed in order of importance based on results of the Weight of Evidence analysis (see [11] for additional details on analysis and results).

| **Number** | **Variable** | **Data (Source**^[[2]](#footnote-2)^**)** | **Variable data type** | **Reference** |
| --- | --- | --- | --- | --- |
| 1 | Distance from built-up areas | Built-up areas (MOIN) | Continuous | [12–14] |
| 2 | Average Population growth in sub-district over 5 years prior to t_0_ | Geographic regions (CBS) | Continuous | [15] |
| 3 | Distance from major roads and highways | Major roads and highways (HUGIS) | Continuous | [16] |
| 4 | Population density in sub-district at t_0_ | Geographic regions (CBS) | Continuous | [15] |
| 5 | Land use/cover | Land use and land cover (CBS and INPA) | Categorical Built/Agriculture/Natural | [15,17–19] |
| 6 | Distance from major running streams | Major running streams (HUGIS) | Continuous | [20] |
| 7 | Protection status | Protected areas (INPA) | Categorical (yes/no) | [15,17–19] |

Out of the variables listed in Table 1 we found that distance from existing built-up land was the most influential factor affecting the location of new development: the probability of conversion from open to built-up decreased with distance (particularly above 200 m). In addition, development was more likely to occur in sub-regions that experienced higher levels of population growth in the five years prior to the simulation period and in areas in proximity to main roads.

We assessed model performance by running the simulation for the period of 2003-2007, using the same parameters obtained in the calibration (1998-2003). We compared the simulated and observed maps using a multiple resolution similarity comparison [21,22]. We found that the similarity between the maps of observed and simulated changes exceeded 50% at a resolution of 650 m and reached > 80% at a resolution of 2,050 m (Fig. 3).

**
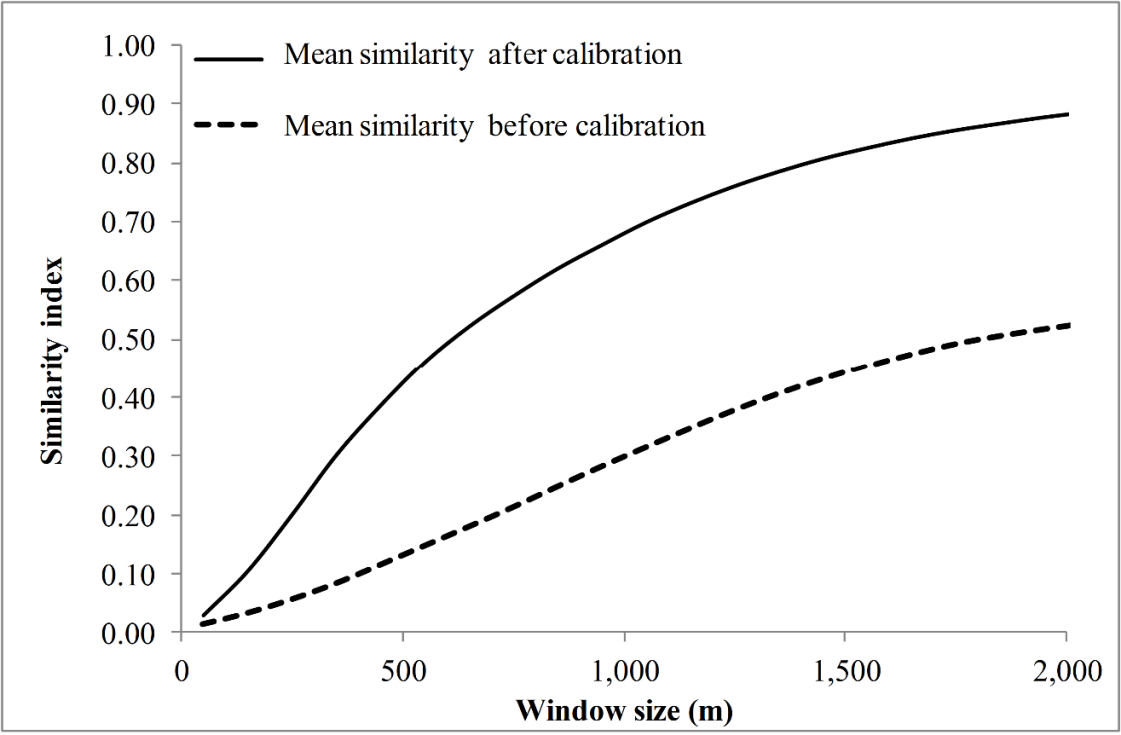
**

Fig. 3 Mean similarity index of observed versus simulated changes over multiple window sizes for the urban growth model before and after calibration.

### **Scenarios**

We constructed two scenarios of urban development, based on studies that were conducted in the study area.

#### **Scenario 1: Regulated development**

This scenario is driven by a growth management policy and assumes that future development and land uses will be determined by the policies suggested in the National Outline Plan 35 (NOP 35) [18].This plan has been in effect since 2005. It includes a growth management policy involving a number of development restrictions, such as requiring new urban development to take place in adjacency to existing urban built-up areas, concentrating development in defined areas, intensifying existing built-up areas, and enforcing minimal density levels in new areas of development [18,23]. According to Frenkel [7, p. 359]: “*This policy was manifested by normative goals for population spatial distribution. In order to generate efficient differential use of land in the various areas, binding development restrictions were set in the national plans. These restrictions included, among others, the obligation to attach any new urban development to an existing urban built-up area; the concentration of development in defined areas; a minimum urban- density requirement in the new built-up areas, as well as the intensification of the old built-up areas*”. In order to simulate this scenario, we applied the following changes to the reference parameters: (1) decreased the patcher/expander ratio by 50% in order to generate a smaller number of new patches; (2) changed the transition probabilities in each district to reflect the growth rates suggested in Frenkel (2004b), in order to reflect a situation whereby development is guided by NOP 35 and existing built-up areas are intensified; (3) increased the mean size of patches by 150% in order to reduce the abundance and formation of small built-up patches; and (4) replaced the land-use map of the initial year with the map of designated land use according to district outline plans, and set negative weights (*W^+^* = -2.0) for agricultural and natural land uses, and positive higher weights for areas designated for development (*W^+^* = 2.0). This was done in order to reflect a situation whereby land-use zoning is imposed and receives greater weight. Hereinafter we refer to this scenario as ”Regulated”.

#### **Scenario 2: Unregulated development**

The second scenario is based on Alfasi et al. [17], who found large gaps between land uses designated by statutory plans and the actual land uses, and showed that development was not restricted by land-use designations of comprehensive district outline plans. In their words ([17], p. 873): “*The actual case-by-case development gradually erodes land-uses originally allocated for farmland and for nature and scenic landscape, turning them into built areas.*” This scenario reflects a situation in which the guidelines for development in NOP 35 are not followed. In order to simulate this scenario, we made the following changes to the reference parameters: (1) increased the patcher/expander ratio by 50% in order to generate a larger number of new patches; (2) increased the transition probabilities by 25% in order to represent increased development; (3) decreased mean size of patches by 100% in order to represent a pattern of smaller and more scattered built-up patches; and (4) set the weights of both agricultural and natural land uses to zero in order to reflect a situation in which future development is not influenced by current or designated land use. Hereinafter we refer to this scenario as ”Unregulated”.

For each scenario we performed a set of simulations, by progressively increasing the transition probabilities by 100% each time, up to values which were 8 times higher than those in the scenarios with development extents comparable to the “business as usual” level. Thus, each scenario was modeled over a range of development extents (these series of simulations for each scenario are referred to hereinafter as scenario sets). Each scenario was simulated for 60 time steps (years). The distribution of built-up areas in the initial year is based on the 2007 map of built-up areas.

1. **References**

1. Soares-Filho BS, Pennachin CL, Cerqueria G. DINAMICA - a stochastic cellular automata model designed to simulate the landscape dynamics in an amazonian colonization frontier. Ecol Modell. 2002;154: 217–235.

2. Samocha Y, Litav M, Fine P, Vizel Y. Development rate of woodland trees in the Judea Mountains. La’yaaran. 1980;30: 6–15. (In Hebrew). Available: (In Hebrew)

3. Carmel Y, Kadmon R, Nirel R. Spatiotemporal predictive models of Mediterranean vegetation dynamics. Ecol Appl. 2001;11: 268–280. Available: http://www.esajournals.org/doi/abs/10.1890/1051-0761(2001)011[0268:SPMOMV]2.0.CO;2

4. Kadmon R, Harari-Kremer R. Studying long-term vegetation dynamics using digital processing of historical aerial photographs. Remote Sens Environ. Elsevier; 1999;68: 164–176. Available: http://www.sciencedirect.com/science/article/pii/S0034425798001096

5. Sapir G, Carmel Y. Predicting revegetation after fire in planted pine forests. Ecol Environ. 2010;3: 14–23. (In Hebrew).

6. IPCC. Climate Change 2007: Synthesis Report. Contribution of Working Groups I, II and III to the Fourth Assessment Report of the Intergovernmental Panel on Climate Change. Core Writing Team, Pachauri R, Reisinger A, editors. Geneva, Switzerland; 2007.

7. IPCC. Special Report on Emission Scenarios. Nakicenovic N, Swart R, editors. Cambridge, UK: Cambridge University Press; 2000.

8. Gea-Izquierdo G, Viguera B, Cabrera M, Cañellas I. Drought induced decline could portend widespread pine mortality at the xeric ecotone in managed mediterranean pine-oak woodlands. For Ecol Manage. Elsevier B.V.; 2014;320: 70–82. doi:10.1016/j.foreco.2014.02.025

9. Allen CD, Macalady AK, Chenchouni H, Bachelet D, McDowell N, Vennetier M, et al. Forest Ecology and Management A global overview of drought and heat-induced tree mortality reveals emerging climate change risks for forests. For Ecol Manage. 2010;259: 660–684. doi:10.1016/j.foreco.2009.09.001

10. Bonham-Carter GF. Geographic Information Systems for Geoscientists: Modelling with GIS. New York, NY: Pergamon; 1994.

11. Troupin D, Carmel Y. Landscape patterns of development under two alternative scenarios: Implications for conservation. Land use policy. Elsevier Ltd; 2016;54: 221–234. doi:10.1016/j.landusepol.2016.02.008

12. Benguigui L, Czamanski D, Marinov M. City Growth as a Leap-frogging Process: An Application to the Tel-Aviv Metropolis. Urban Stud. 2001;38: 1819–1839. doi:10.1080/00420980120084877

13. Benguigui L, Czamanski D. Simulation Analysis of the Fractality of Cities. Geogr Anal. 2004;36: 69–84.

14. Benguigui L, Czamanski D, Marinov M. The dynamics of urban morphology: the case of Petah Tikvah. Environ Plan B Plan Des. 2001;28: 447–460. doi:10.1068/b2703

15. Orenstein DE, Hamburg SP. Population and pavement: population growth and land development in Israel. Popul Environ. 2010;31: 223–254. doi:10.1007/s11111-010-0102-4

16. Levin N, Lahav H, Ramon U, Heller a, Nizry G, Tsoar a, et al. Landscape continuity analysis: A new approach to conservation planning in Israel. Landsc Urban Plan. 2007;79: 53–64. doi:10.1016/j.landurbplan.2006.04.001

17. Alfasi N, Almagor J, Benenson I. The actual impact of comprehensive land-use plans: Insights from high resolution observations. Land use policy. Elsevier Ltd; 2012;29: 862–877. doi:10.1016/j.landusepol.2012.01.003

18. Frenkel A. The potential effect of national growth-management policy on urban sprawl and the depletion of open spaces and farmland. Land use policy. Elsevier; 2004;21: 357–369. doi:10.1016/j.landusepol.2003.12.001

19. Frenkel A. A land-consumption model: Its application to Israel’s future spatial development. J Am Plan Assoc. 2004;70: 453–470. doi:10.1080/01944360408976394

20. Maruani T, Amit-Cohen I. The effectiveness of the protection of riparian landscapes in Israel. Land use policy. 2009;26: 911–918. doi:10.1016/j.landusepol.2008.11.002

21. Almeida CM, Gleriani JM, Castejon EF, Soares-Filho BSS. Using neural networks and cellular automata for modeling intra-urban land use dynamics. Int J Geogr Inf Sci. 2008;22: 943–963. doi:10.1080/13658810701731168

22. Soares-Filho BS, Rodrigues HO, Costa WL. Modeling Environmental Dynamics With DINAMICA EGO [Internet]. Belo Horizante/Minas Gerais, Brazil; 2009. Available: www.csr.ufmg.br/dinamica.

23. Assif S, Shachar A. TAMA 35 - Ikarei H’Tokhnit (NOP 35 - plan highlights). Jerusalem, Israel. (In Hebrew): Planning Authority - Israel Ministry of Interior; 2005.

1. Built-up areas are based on the data cited in S2. They include man-made development such as structures and buildings etc. They do not include infrastructure such as roads or powerlines. [↑](#footnote-ref-1)
2. Abbreviations of sources: MOIN – Israel Ministry of Interior; CBS – Israel Central Bureau of Statistics; HUGIS – Hebrew University of Jerusalem GIS Center; INPA – Israel Nature and Parks Authority. [↑](#footnote-ref-2)
